# Transplantation Improves Patient Survival in a PD-first Program in South Africa

**DOI:** 10.1097/TXD.0000000000001914

**Published:** 2026-03-17

**Authors:** Kathryn Manning, Robert Freercks, Mogamat Razeen Davids, Berzelius Klein-Motsumi, Andrew D. Redd, Jason Ensor, Lungiswa Mtingi-Nkonzombi, Elmi Muller

**Affiliations:** 1 Department of Surgery, University of Cape Town, Faculty of Health Sciences, Groote Schuur Hospital, Cape Town, South Africa.; 2 Department of Medicine, Nelson Mandela University, Livingstone Hospital, Gqeberha, South Africa.; 3 Division of Nephrology, Stellenbosch University, Faculty of Medicine and Health Sciences, Cape Town, South Africa.; 4 Department of Internal Medicine, Faculty of Health Sciences, Walter Sisulu University, Nelson Mandela Academic Hospital, Mthatha, South Africa.; 5 Division of Intramural Research, NIAID, NIH, Bethesda, MD.; 6 Department of Pathology, Institute of Infectious Disease and Molecular Medicine, University of Cape Town, Cape Town, South Africa.; 7 Department of Medicine, Johns Hopkins University, Baltimore, MD.; 8. Faculty of Medicine and Health Sciences, Stellenbosch University, Cape Town, South Africa.

## Abstract

**Background.:**

There are limited data on kidney replacement therapy (KRT) allocation and outcomes in patients in kidney failure (KF) who access public healthcare in South Africa.

**Methods.:**

This retrospective cohort study included patients referred for KRT at the time of KF diagnosis. Incident KF cases were identified between 2012 and 2020, followed from referral until death, kidney transplantation, or continued waitlisting at study end (December 31, 2023). Descriptive analyses and comparisons were performed between KRT allocation and outcomes. Time-to-event analyses employed competing risk models to estimate cumulative incidence functions, whereas Kaplan-Meier methods were applied to calculate survival probabilities.

**Results.:**

Overall, 761 patients were referred with KF, of which 598 (79%) were untreated and presumed to have died. Untreated patients were either not considered at referral (n = 432), or not accepted at KRT committee meeting (n = 175) because of policy-driven factors. Of those presented to the KRT committee (n = 338), 48% (n = 163) were accepted onto the dialysis program and waitlisted for transplantation. Accepted patients were younger and had greater medical stability and socioeconomic circumstances compared with non-accepted patients. Only 21% (n = 34) of patients initiated on dialysis were transplanted. At 5 y post-KRT initiation, there was a greater probability of dying on the waitlist compared with receiving a transplant (cumulative incidence function 35% [95% CI, 27-42] versus 18% [95% CI, 13-25]), and post-transplantation survival was significantly greater than pre-transplant survival (100% versus 61% [95% CI, 53-69]).

**Conclusions.:**

Our study findings align with the challenges of providing dialysis and transplantation in a lower- to middle-income setting where patients were most often precluded from KRT because of poorly controlled comorbidities or a lack of unit capacity. There was a clear survival advantage in patients who were transplanted over those who remained on dialysis; however, transplant services remain limited.

## INTRODUCTION

On a global scale, chronic kidney disease (CKD) is a growing public health burden^1,2^ and is forecast to be the fifth leading cause of death by 2040.^3^ The estimated prevalence of CKD in South African cohorts has ranged between 6% and 17%,^4-7^ yet the true burden of kidney failure (KF) is unknown. Current estimates are based on the South African Renal Registry that includes treated KF, that is, patients registered on dialysis or transplanted.^8^ Yet, this is considered to be the “tip of the iceberg” in the prevailing CKD burden in the public sector because estimates exclude unreferred patients in KF and patients not accepted onto KRT programs.

Public-sector kidney replacement therapy (KRT) access in the Eastern Cape provides a compelling model for understanding challenges across Africa. Ninety-three percent of the population are medically uninsured; thus, the CKD burden falls disproportionately on underresourced public healthcare facilities. The province reflects many of the structural realities of lower- and middle-income countries (LMICs): a large population with high KF burden, but very limited public KRT capacity. As in much of Africa, available services reach only a fraction of those in need. South African legislation mandates that private medical insurance covers dialysis, creating a stark contrast in treatment rates between sectors: a study in Eastern Cape reported rates of 49 per million population (pmp) in the public sector versus 1435 pmp in the private sector.^9^ This divergence illustrates the inequities in access that are also characteristic of African health systems, where small, well-resourced populations have access to advanced care, whereas the majority remain underserved.^10-12^ In the Eastern Cape, most KRT is delivered via dialysis, with transplantation dependent on referral to the neighboring Western Cape. This reliance on out-of-province transplant services mirrors the regional and cross-border dependencies common across Africa, where specialist centers are sparse and unevenly distributed.

In South Africa and other LMICs, risk factors for CKD are increasing rapidly in younger and middle-age population groups.^7,13,14^ In the coming decades, there is concern that the burden could debilitate a substantial proportion of the economically active population.^11^ Although national data on the incidence of CKD are lacking, the substantial rise in the rate of treated KF observed in the private sector in the pre-COVID era^15^ suggests that the true burden of KF in the public sector could be equivalent—if not worse—in a population that relies on public healthcare services with strict resource rationing of KRT.^16^ Although public healthcare is free for uninsured patients, it comes with many trade-offs such as longer waiting times for medical assessments and limited treatment options for delaying CKD progression. In the event of a KF diagnosis, a minority of patients are eligible for KRT if deemed a good transplant candidate and if there is sufficient local resources.^16,17^ For example, 2 South African studies in other provinces have shown KRT allocation rates of 30%^18^ to 46%^19^ of referred patients in KF. However, there are inequalities in provision of KRT whether by dialysis and/or kidney transplantation exist within and between provinces.^10,20^

Healthcare policies should be informed by studies on mortality from both treated and untreated KF, waitlist survival and median time to transplant, yet these epidemiology estimates remain limited in South Africa. Supportive palliative care is offered to referred patients with KF who are not accepted for KRT; however, the true magnitude of this population remains unclear. Survival outcomes in patients who received KRT (dialysis and/or transplant for public and private patients) have been reported in a national study 90.4% at 1 y, and observed an increased risk of death in Eastern Cape province compared with the reference province (Western Cape).^21^ Another KRT unit reported 5-y survival at 57%; however, the data spanned from 1969 to 2015,^22^ and survival has improved in recent decades. For kidney transplantation, 5-y patient and graft survival have been reported as 83%–88% and ~80%, respectively, in South African units.^23,24^ However, it remains unclear whether there is a survival advantage of transplantation over dialysis in public healthcare settings.

This study aimed to describe the characteristics, allocation and survival outcomes of patients referred for rationed KRT in a resource-constrained public sector center in the Eastern Cape Province of South Africa. The study also aimed to provide estimates for regional prevalence of treated KF, survival and probability that account for competing risks.

## MATERIALS AND METHODS

### Study Design, Cohort, and Setting

We conducted a retrospective review of patients with KF who were referred and presented for KRT consideration at Livingstone Hospital in Gqeberha between March 2012 and March 2020. Patients were followed up from the date of presentation until December 31, 2023. Inclusion criteria for patients in this study were ≥18 y of age, stage 5 CKD (estimated glomerular filtration rate [eGFR] <15) and were formally referred for consideration of KRT.

Livingstone Hospital is a public sector hospital providing clinical care to predominantly uninsured patients that services the Western region the Eastern Cape and serves approximately 1.6 million people who reside in the Sarah Baartman and Nelson Mandela Bay Districts.^25^ The KRT program is a state-provided system where patients accepted for KRT receive full access to KRT at no personal cost.

### KRT Program and Allocation

Patients with CKD stage 5 who were considered eligible for KRT (eGFR < 15 and candidates for transplantation) were referred to the nephrology department at Livingstone Hospital for assessment. Patients were then presented at a KRT committee meeting that includes nephrologists, physicians, hospital managers, a social worker, psychologist and nursing staff who jointly assessed eligibility and feasibility of initiating KRT. A pre-requisite for allocation of KRT is a patient’s eligibility for kidney transplantation.^17^ The prioritization of KRT is assessed against the KRT criteria and each patient is allocated to 1 of 3 categories^16,17^ (**Figure S1, SDC**, https://links.lww.com/TXD/A833). Reasons for non-acceptance to KRT are primarily medical, social or resource related. Patients are also assessed for uncontrolled comorbidities and/or progressive target organ damage (TOD), smoking, habitual drug or alcohol use, and/or socioeconomic circumstances unconducive to successful posttransplant care.

Category 3 patients are not offered KRT because of low eligibility for transplantation and are not accepted into the program if 1 exclusion criterion is met. Common factors include older age, uncontrolled HIV, and advanced TOD. Category 2 patients with controlled comorbidities are allocated dialysis providing that space and human resources are available, whereas category 1 patients (usually very fit and young patients without comorbidities or social exclusions) are mandated to receive dialysis. Category 3 and 2 patients who are not offered KRT (also referred to as “untreated KF”) are referred for supportive and palliative care at their local hospital or clinic.^26^ For accepted patients, the unit provides a peritoneal dialysis (PD)-first program using a structured policy approach in which peritoneal dialysis is designated as the first-line modality for KRT, with hemodialysis (HD) offered only when PD is contraindicated or not feasible. Contraindications for PD include visual impairment, poor social circumstances, or lack of space to store PD supplies. All patients are waitlisted for transplant on commencement of dialysis.

### Data Collection and Management

Patient data were retrieved from archived patient records, laboratory results, and written summaries of KRT presentation meetings. Data were collected and managed on the Research Electronic Data Capture platform.^27^ All patient records were anonymized using a unique patient study number. Data were collected at KRT presentation and at end of study as lost to follow-up (LTFU), death, or alive on December 31, 2023. Sociodemographic data included age at presentation, sex, marital status, and social variables such as employment, access to water and sanitation amenities, social support, and geographical location. Data on clinical variables included CKD etiology, comorbidities (including but not limited to obesity, diabetes, hypertension, hepatitis B and C, HIV, tuberculosis, metastatic cancers and connective tissue disease), and creatinine and estimated GFR (CKD-Epi)^28^ at the time of presentation. Program-based data included category allocation and reasons for non-acceptance onto KRT program per prioritization criteria. There were incomplete records for category 3 patients referred but not presented to the committee because data were kept in medical records at the primary referring hospital. All patients who were presented to the committee had complete and detailed records on acceptance and nonacceptance.

### Outcomes

The primary outcomes of interest were to (1) quantify the proportion of referred patients with KF who received KRT over study period, (2) determine the KRT treatment rate by modality (pmp) for the Western region of the Eastern Cape, (3) describe KRT allocation and modality switches, and (4) calculate survival estimates for waitlisted patients who remained on dialysis versus those receiving transplantation.

### Data Analysis

Descriptive statistics were used to summarize the cohort. Medians with interquartile range (IQR) were presented for continuous variables and frequency and percentage for categorical variables. Data were described and compared between those not accepted for KRT versus those accepted for KRT. The Wilcoxon rank-sum test and the Kruskal-Wallis test were used for two and three-sample comparisons respectively. Chi-squared or Fisher exact test was used for categorical comparisons, as appropriate.

Only patients who were established on dialysis for >90 d were included in the analysis. Four patients were excluded from analysis on outcomes because of death before starting dialysis. The prevalence of KRT was calculated at the study mid-point (year 2016) from the number of patients on KRT on December 31, 2016, divided by the mid-year regional population estimate^25^ of the uninsured population^29^ in 2016. Probability of death on waitlist or transplant was compared using the cumulative incidence competing risk and Kaplan-Meier methods to assess over or underestimation of risks as described by van Geloven et al.^30^ In the Fine and Gray’s proportional subhazards model, death and transplant were the outcomes of interest and competing events, respectively. Median waitlist time (defined as time at which 50% of patients received a transplant during the study period) was not calculated using the competing risks method since fewer than half of the total patients were transplanted. Median follow-up time on dialysis until death, transplant or end of study was calculated and presented as median with IQR. For descriptive and comparability purposes, the Kaplan-Meier method was used to present probability of death or transplant, and pre- and posttransplant survival. Finally, in a the small subset of patients who did receive transplant (n = 34), time to death or graft failure was estimated using the Kaplan-Meier method from day 1 of transplant to the respective outcome. Patients alive on December 31, 2023, regaining renal function or LTFU were censored.

### Ethical Considerations

Ethics approval was obtained from University of Cape Town Human Research Ethics Committee (045/2024).

## RESULTS

### Allocation of KRT in Referred Patients With KF

**FIGURE 1.**
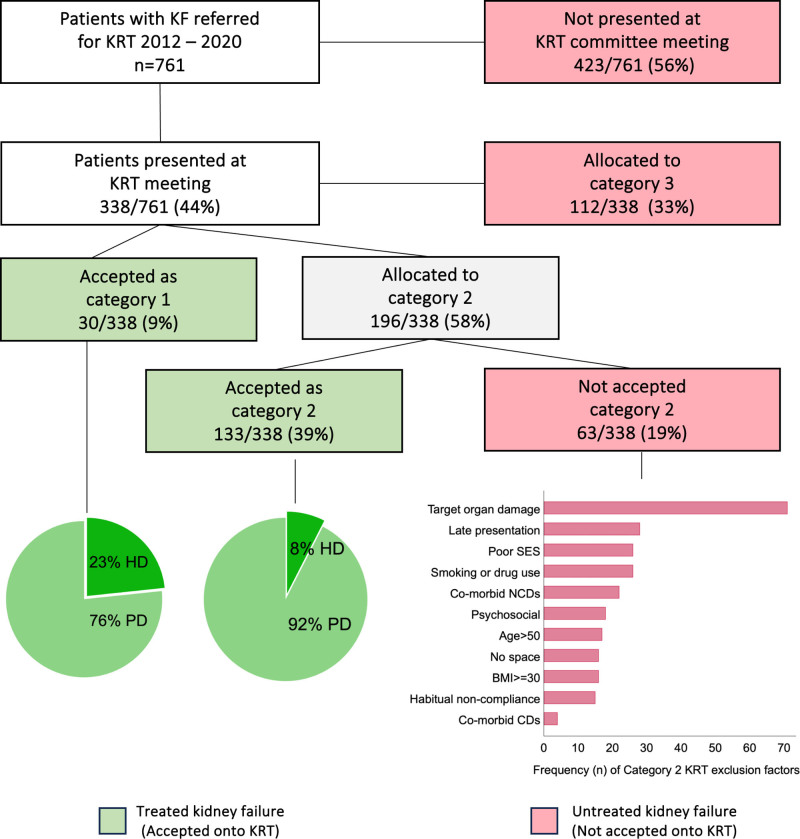
Patient flow from referral to KRT allocation and reasons for nonacceptance. BMI, body mass index; CDs, communicable diseases; HD, hemodialysis; KF, kidney failure; KRT, kidney replacement therapy; NCDs, noncommunicable diseases; PD, peritoneal dialysis; SES, socioeconomic status.

From February 2012 to February 2020, 761 incident KF patients were referred to the livingstone hospital renal unit for consideration of KRT (Figure 1). Of these, 79% (598/761) of patients did not receive KRT; 423 patients were referred with 1 or more category 3 factors and 175 presented patients were not accepted for KRT because of category 2 and 3 factors after committee review. Untreated patients in KF were allocated palliative and supportive care as per national guidelines.^26^ Where documented, the most common category 3 factors for nonacceptance (**Figure S1, SDC**, https://links.lww.com/TXD/A833) were advanced organ failure, habitual nonadherence with medical treatment, uncontrolled HIV, obesity, and older age with diabetes. Category 2 patients presented to the committee but not accepted were because of medical, social, and/or psychosocial factors, including TOD, late presentation, poor socioeconomic status, smoking or drug use, poorly controlled noncommunicable diseases (NCDs) and comorbidities (such as poorly controlled hypertension and diabetes mellitus), and unfavorable psychosocial factors. Comorbid communicable diseases including hepatitis B (n = 3) and uncontrolled HIV (n = 1) were less frequently observed because uncontrolled HIV is considered an independent category 3 exclusion factor. Even though 87% were on antiretroviral therapy at time of presentation, only 48% were virally suppressed.

The remaining 163 patients (21%) were accepted in either category 1 (n = 30) or category 2 (n = 133), and allocated to PD unless contraindicated. Treated patients were more likely to be married, employed and living in formal housing, and reflected the clinical eligibility criteria for KRT, as they were younger, more educated, and had lower prevalence of NCDs and risk factors for NCDs compared with patients not accepted (Tables 1 and 2). Fourteen percent were HIV positive and virally suppressed on ART.

**TABLE 1.** Comparison of sociodemographic characteristics of presented^*a*^ patients with KF between those referred for and accepted or not accepted for KRT

	Total	Accepted onto KRT	Not Accepted onto KRT	*P*
	N = 338	N = 163 (48.2%)	N = 175 (51.8%)	
Age at presentation, y	38 (29–46)	35 (29–43)	40 (31–48)	0.001
Sex, female	163 (48)	75 (46)	88 (50)	0.430
Highest school grade completed	11 (9–12)	11 (10–12)	10 (8–11)	0.001
Employed	134 (40)	76 (47)	58 (33)	0.011
Tertiary education				
Completed/enrolled	43 (13)	26 (16)	17 (10)	0.086
No tertiary education	295 (87)	137 (84)	158 (90)	
Married	95 (28)	56 (34)	39 (22)	0.014
Dependents	224 (66)	115 (71)	109 (62)	0.220
Currently Smoking	73 (22)	22 (13)	51 (29)	<0.001
Consuming alcohol	91 (27)	41 (25)	50 (29)	0.480
Illicit substance abuse	19 (6%	4 (2)	15 (9)	0.015
Toilet facilities				
Inside toilet	274 (81)	133 (82)	141 (81)	0.390
Outside toilet	62 (18)	30 (18)	32 (18)	
Pit Latrine	2 (1)	0 (0)	2 (1)	
Water access				
Inside tap	298 (88)	145 (89)	153 (87)	0.660
Outside tap	40 (12)	18 (11)	22 (13)	
Housing				
Formal dwelling	308 (91)	153 (94)	155 (89)	0.087
Informal dwelling	30 (9)	10 (6)	20 (11)	

Data are presented as median (IQR) for continuous measures, and n/total (%) for categorical measures.

^*a*^ Data include only category 1 and 2 patients who were presented for KRT (n = 338) and do not include patients referred and not referred (Figure 1).

IQR, interquartile range; KRT, kidney replacement therapy.

**TABLE 2.** Clinical characteristics of patients presented^*a*^ patients with KF between those referred for and accepted or not accepted for KRT

	Total	Accepted onto KRT	Not Accepted onto KRT	*P*
	N = 338	N = 163	N = 175	
Serum creatinine, μmol/L	935 (639–1441)	1000 (651–1534)	894 (626–1400)	0.240
eGFR (CKD-EPI), mL/min/1.73 m^2^	5 (3–8)	5 (3–8)	5 (3–8)	0.490
Hemoglobin, g/dL	8 (7–9)	8 (7–9)	8 (7–9)	0.470
BMI classification^*b*^				0.210
Normal weight	134/312 (43)	72/155 (46)	62/157 (39)	
Overweight/obese	178/312 (57)	83/155 (54)	95/157 (61)	
SBP, mm Hg	148 (127–180)	144 (123–179)	156 (131–180)	0.047
DBP, mm Hg	88 (80–100)	83 (80–97)	90 (79–100)	0.180
HIV positive	46 (14)	23/163 (14)	23 (13)	0.800
On ART	43/46 (93)	23/23 (100)	20/23 (87)	0.300
HIV RNA <200 copies/mL	34/46 (74)	23/23 (100)	11/23 (48)	<0.001
Hypertension	295 (87)	135 (83)	160 (91)	0.018
Diabetes	58 (17)	15 (9)	43 (25)	<0.001
SLE	12 (4)	10 (6)	2 (1)	0.013
Hepatitis B	18 (5)	9 (6)	9 (5)	0.880
Previous tuberculosis	51 (15)	26 (16)	25 (14)	0.670
Primary etiology of KD				
Hypertension	229 (68)	94 (58)	135 (77)	<0.001
Diabetes mellitus	54 (16)	14 (9)	40 (23)	<0.001
Glomerulonephritis	55 (16)	40 (25)	15 (9)	<0.001
Other genetic	17 (5)	9 (6)	8 (5)	0.690
HIVAN	13 (4)	8 (5)	5 (3)	0.330
ADPKD	12 (4)	7 (4)	5 (3)	0.560
Lupus nephritis	11 (3)	9 (6)	2 (1)	0.030
Other	9 (3)	3 (2)	6 (3)	0.500
FSGS^*c*^	4 (1)	2 (1)	2 (1)	
Obstructive uropathy^*c*^	6 (2)	3 (2)	3 (2)	
Unknown^*c*^	1 (0)	0 (0)	1 (1)	

Data are presented as median (IQR) for continuous measures, and n/total (%) for categorical measures.

^*a*^ Data includes only category 1 and 2 patients who were presented for KRT (n = 338).

^*b*^ N varies because of missing data.

^*c*^ FSGS, obstructive uropathy, and unknown: Samples sizes too small for meaningful comparison.

ADPKD, autosomal-dominant polycystic kidney disease; ART, antiretroviral therapy; BMI, body mass index; CKD-Epi, Chronic Kidney Disease Epidemiology Collaboration Equation; DBP, diastolic blood pressure; eGFR, estimated glomerular filtration rate; FSGS, focal segmental glomerulosclerosis; IQR, interquartile range; HIVAN, HIV-associated nephropathy; HIVVL, HIV viral load; KRT, kidney replacement therapy; SBP, systolic blood pressure; SLE, systemic lupus erythematosus.

**FIGURE 2.**
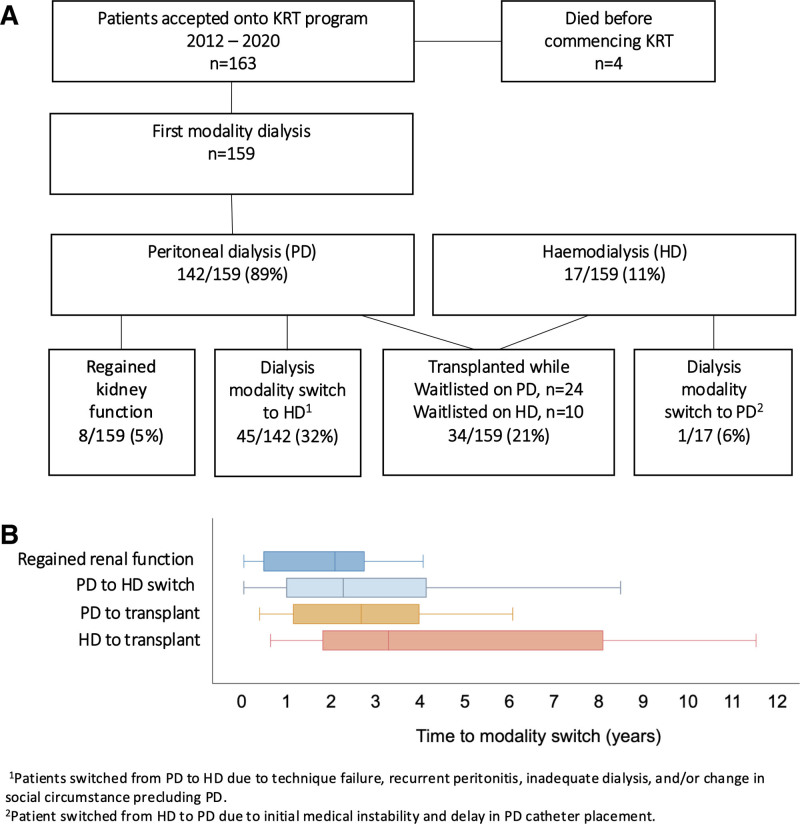
Flow and timing of patients from first modality to modality switch. A, Patients first modality to regained kidney function, mode of dialysis switch and transplant time (B) on KRT. HD, hemodialysis; KRT, kidney replacement therapy; PD, peritoneal dialysis.

Patients established on dialysis were followed up from the day of initiation to death, transplant, LTFU, or end of study period (December 31, 2023) for a median of 5.2 y (IQR, 2.7–7.2 y). Four patients died before commencing dialysis with the remaining 159 followed from initiation of dialysis to death (Table 3). Most patients (n = 142, 89%) were initiated on PD compared with HD (Figure 2A). Eight patients commenced on PD with a median eGFR of 5.0 mL/min/1.73 m^2^ (IQR, 3.5–11.0 mL/min/1.73 m^2^) at referral regained kidney function (Figure 2B). Kidney function recovery was between 0.5 and 4 y after initiation of dialysis. Five patients had severe malignant hypertension as a cause of KF, which slowly resolved over an extended period with antihypertensive treatment and optimal blood pressure control. Among the remaining patients, causes of KF were HIV-associated nephropathy, Crescentic membranoproliferative glomerulonephritis, and lupus nephritis (LN Class III/ IV), which also improved with treatment. Switching from PD to HD occurred in 32% of patients (n = 45/142). One patient started on HD because of medically instability and delayed in catheter insertion but switched to PD thereafter (Figure 2A).

**TABLE 3.** Outcomes of patients accepted for KRT

	n/N (%)	Time from KRT initiation to outcome (y), median (IQR)
Waitlisted on dialysis	159	5.25 (2.67–7.22)
Died before starting KRT	4/163 (2)	
Regained renal function	8/159 (5)	2.09 (0.48–2.75)
Died on dialysis	66/159 (42)	2.14 (0.71–4.05)
Died on PD (n = 51)	51/66 (77)	2.15 (0.71–4.05)
Infection	13/51 (25)	2.27 (2.10–3.61)
Cardiovascular	10/51 (20)	3.99 (2.41–5.31)
Fluid overlaod with pulmonary edema	9/51 (18)	0.96 (0.46–2.15)
ESKD/failed PD	7/51 (14)	1.27 (0.59–3.02)
Unknown	7/51 (14)	1.89 (1.36–6.34)
Neurological^*a*^	2/51 (4)	2.68
Malignancy^*a*^	2/51 (4)	4.74
Suicide^*a*^	1/51 (2)	0.41
Died on HD (n = 15)	15/67 (22)	3.34 (1.74–4.11)
Infection	5/15 (40)	1.74 (0.74–1.89)
ESKD/failed HD	4/15 (27)	3.66 (2.85–3.97)
Cardiovascular^*a*^	3/15 (20)	4.10
Pulmonary edema^*a*^	1/15 (7)	0.40
Suicide^*a*^	1/15 (7)	3.22
Malignancy^*a*^	1/15 (7)	6.09
Transplanted	34/159 (21)	2.67 (1.32–4.53)
Donor type		
Living-related donor	15/34 (44)	1.81 (0.98–3.28)^*c*^
Deceased donor	19/34 (56)	4.08 (1.51–5.85)^*c*^
Died posttransplant	1/34 (3)	1.14^*d*^
Graft failure	2/34 (6)	4.97^*d*^
TB post-KRT initiation^*b*^	23/159 (14)	—

Median time on KRT was calculated from start of dialysis until death, or graft failure if transplanted, or end of study period if alive (December 31, 2023).

^*a*^ n too small for distributional measures, that is, IQR.

^*b*^ Date of TB diagnosis not recorded thus time to infection could not be calculated.

^*c*^ Time calculated from dialysis start to transplant.

^*d*^ Time calculated from transplant date to death or graft failure.

ESKD, end stage kidney disease; HD, hemodialysis; IQR, interquartile range; KRT, kidney replacement therapy; PD, peritoneal dialysis; TB, tuberculosis.

### KRT Prevalence Rate

The KRT prevalence rate for the Western region public sector was calculated at the mid-point of the study (on December 31, 2016) as 122 pmp. The unit was providing dialysis to 120 patients and 78 patients were attending the transplant clinic with a functioning kidney graft. The individual prevalence rates of PD, HD, and transplant were 31, 43, and 48 pmp, respectively.

### Cumulative Incidence of Death and Kidney Transplantation

Cumulatively, 42% (66/159) of patients died while waitlisted on dialysis: 51 (77%) on PD versus 15 (23%) on HD. In both modalities, most deaths were attributed to infection, cardiovascular factors, pulmonary edema, technique failure, and/or inadequate dialysis. Overall median time to death on dialysis was 2.14 y (IQR, 0.71–4.05 y); however, patients on PD had a shorter time to death compared with patients on HD (median 2.15 versus 3.34 y). Twenty-one percent (34/159) received a transplant within the study period (2012–2023) (Table 3). Of these, most transplant patients were on PD (24/34, 71%), and the majority transplanted within 4 y of dialysis initiation (Figure 2B). HD patients (10/34, 29%) showed longer waiting times and a larger variation in time to transplantation compared with patients initiated on PD. Median time to transplant was 2.67 y (IQR, 1.32–4.53 y); however, patients who received a transplant from living-related donors waited significantly shorter (median 1.8 y) compared with patients who were transplanted with deceased donor kidney (median 4.1 y) (Table 3).

**FIGURE 3.**
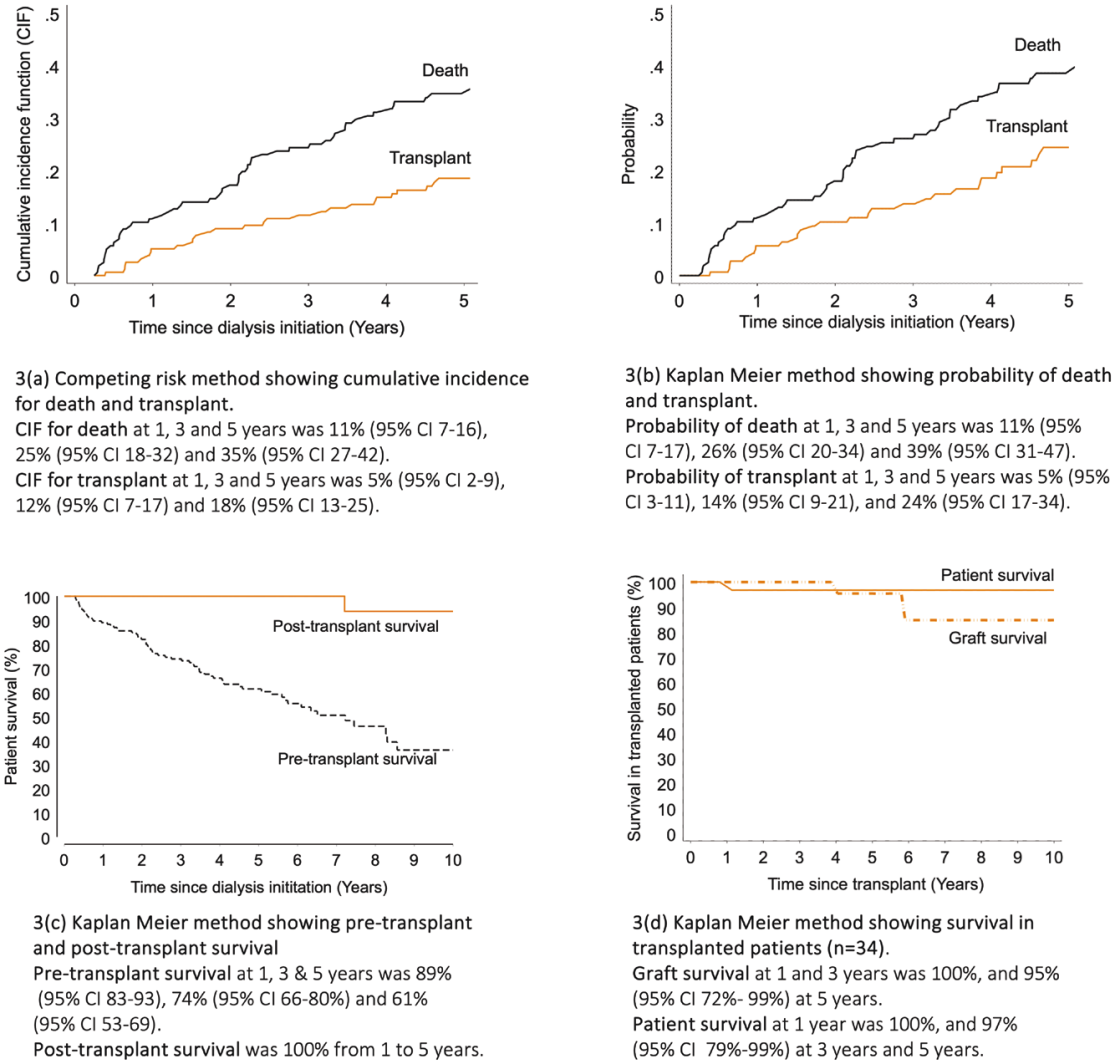
Cumulative incidence and survival estimates for patients who received KRT. KRT, kidney replacement therapy.

Figure 3A presents the competing risks method for cumulative incidence of dying on dialysis before receiving a transplant as 11%, 25%, and 35% at years 1, 3, and 5. Probability of transplant was lower at 5% (95% CI, 2-9) at 1 y, 12% (95% CI, 7-17) at 3 y, and 18% (95% CI, 13-25) at 5 y. Kaplan-Meier probability estimates (Figure 3B) were similar at 1 and 3 y yet diverge at later time points [at 5 y: competing risk method estimate of 18% (95% CI, 13-25) versus Kaplan-Meier estimate 24% (95% CI, 17-34)]. There were very few significant differences between outcomes groups at 5 y post dialysis initiation; a greater proportion of patients who died or were transplanted patients were married, and although there was a moderate association between diabetes and death (*P* = 0.053), the sample sizes were too small for meaningful interpretation (**Table S1, SDC,** https://links.lww.com/TXD/A832).

Pretransplant survival (survival on dialysis up until transplantation) was 89%, 74%, and 61% at years 1, 3, and 5, respectively. Posttransplant survival was significantly higher for those who were initiated on dialysis and subsequently received a transplant [100% survival at years 1, 3, and 5 (Figure 3C)]. Only marital status differed significantly between transplanted and nontransplanted patients (*P* = 0.018) (**Table S2, SDC,** https://links.lww.com/TXD/A832). Death-censored graft survival was 100% at 1 and 3 y, and 95% (95% CI, 72%-99%) at 5 y and patient survival was 100% at 1 y, and 97% (95% CI, 79%-99%) at 3 and 5 y (Figure 3D). Only 1 patient died with a functioning graft of an unknown cause at 1.1 y posttransplant, and 2 patients lost their grafts at around 4 and 6 y posttransplantation.

## DISCUSSION

This is the first study in South Africa to follow public-sector patients from KF diagnosis to death or transplant. Our findings align with observations from many LMIC healthcare settings, which show a prominent treatment gap between treated and untreated KF. This gap is likely because of rising CKD prevalence amid stagnant dialysis capacity and transplantation rates in the South African public healthcare sector. Most patients in KF could not access KRT and received conservative care until death. In contrast, highly selected patients allocated to dialysis and the transplant waitlist were more likely to die on dialysis than receive a transplant.

The large treatment gap, that is, 4 of every 5 patients with KF who required KRT were not prioritized to receive it, is a novel finding of this study as it provides greater context on the true burden of KF. Current estimates of KF rely on the South African Renal Registry, which estimate incidence and prevalence rates of patients registered on dialysis or transplanted,^8^ but does not capture patients referred and not accepted. Efforts have been made to measure the burden of CKD, KF, and KRT in African countries using renal registries^2,31,32^; however, these estimates likely underrepresent true burden of KF by excluding data on patients who similarly could not access KRT. Our treatment gap (21% treated versus 79% untreated) aligns with estimates from Liyanage et al^33^ who reported African KRT access rates of 9%–16%—the lowest in the world—compared with Asia (17%–34%), Europe (33%–70%), Oceanias (41%–83%), Latin America (48%–93%), and North America (~95%). This is further supported by the Global Burden of Disease study on CKD, which found that sub-Saharan Africa had the lowest ratio of KF replacement therapy to advanced CKD.^31^ More recently, the 2023 ISN Global Kidney Health Atlas for the Africa Region reported similar observations regarding the KRT treatment gap, attributing it to low GDP with subsequent poor health spending, insufficient healthcare resources to detect and treat KF, poor data capture on epidemiology of CKD, and a lack of commitment to implement primary and secondary prevention strategies.^32^

For uninsured KF patients, the probability of receiving life-sustaining KRT is influenced by several factors that precede referral. Patients who presented late often had minimal or no residual kidney function, multiple risk factors for CKD and already had signs of TOD. Alternatively, patients allocated to KRT reflected the national KRT policy that prioritizes younger patients with controlled NCDs and infectious diseases to ensure the greatest chance of transplantation and long-term survival.^17^ Our treatment rate (prevalence of treated KF: 122 pmp) aligned more closely with the global estimate range for lower-income countries and LMICs (4–499 pmp).^2^ In contrast, the treated KF rate for medically insured patients in the Eastern Cape’s private sector were recently reported as 1141 pmp,^34^ in line with rates for middle- to high-income countries (610–1009 pmp)^2^. These rates are indicative of a more universal KRT policy with expanded acceptance criteria that can accommodate older patients with underlying comorbidities.

In South Africa, CKD risk factors are increasing at a concerning rate. Over the last 2 decades, the country has focused primarily on the HIV and tuberculosis disease epidemics without the equivalent effort in NCD prevention and management.^35^ These epidemics have now converged and are significantly contributing to the CKD burden.^36,37^ This was evident in our study in that despite the relatively younger age of referred patients, we still observed a high prevalence of HPT, advanced DM, overweight/obesity, and hypertensive TOD in excluded patients <50 y of age.

Our analysis strongly supports improved survival outcomes in transplanted patients. Five-year pretransplant (dialysis only) and posttransplant survival was 61% versus 100%, respectively. The transplanted cohort (n = 34) also showed patient and graft survival rates ≥95% at 5 y posttransplant. Although 1-y pretransplant survival (89%) is comparable to the national estimate for South Africa (90%), the latter includes both private and public sector patients.^21^ At 5-y postinitiation of dialysis, competing risks analysis showed a higher probability of dying (35%) while on dialysis (if not transplanted in that time) than receiving a transplant (18%). Although limited international studies have found similar outcomes,^38^ others have observed the reverse finding where waitlisted patients have a greater chance of being transplanted than dying on dialysis.^39-42^ Many of the deaths from fluid overload and infection were because of delayed access to healthcare, especially for patients living >100 km from the hospital or those exposed to opportunistic infections. Lower survival among dialysis patients in our cohort also reflects insufficient surgical support structures that are required to provide arteriovenous fistulas in HD, and the limited opportunity for PD patients with technique failure to switch to HD when needed.

The kidney transplantation prevalence rate at the mid-point of our study (2016) was 48 pmp, which lies between the rates reported for LMICs (27 pmp) and upper middle-income countries (83 pmp).^43^ This aligns with our low crude cumulative transplant rate (21%) and the stagnant transplant rates for the province reported by Mtingi-Nkonzombi et al^9^ over a similar study period. Median waitlist time for all patients on dialysis was around 5 y, increasing risk for vascular complications and comorbidities that may subsequently preclude transplantation. Patients without living-related donors, waited around 4 y for a deceased donor transplant—significantly longer than the 1.2 y waiting time in transplanted patients in a US cohort.^44^ Although not the primary focus of this study, the low rate of transplantation and lack of expansion can be explained by the low number of local donors, low donor consent and conversion rates, lack of infrastructure and human resources.^45-48^ Currently, Eastern Cape patients with donors must travel >800 km to the Western Cape for transplant surgery.

Based on our preliminary findings, we recommend that the Eastern Cape prioritizes primary and secondary preventative of CKD, increases the provision of PD in rural areas, and expands transplantation services as the cornerstone of KRT. In addition, there is an urgent need to address the KF treatment gap. The rollout of a PD-first policy is one such strategy that has been successfully implemented in both high-income countries and LMICs, as it is cost-effective and limits need for lengthy travel while acting as a bridge to transplantation.^49,50^ Reducing incident CKD cases and subsequent KF would ease pressure on resource-constrained units, whereas transplanting patients would experience an increase in quality of life, life expectancy, and open up dialysis slots for other KF patients.^51,52^ Widespread public and health sector education campaigns are foundational. International centers with developed economies have implemented interventions to support organ donation through educational opt in and out programs,^53,54^ but these may be ethically and legally problematic in South Africa.^55^ Although this study has added to the limited knowledge on a public health rationed KRT program in a resource-constrained province, a more accurate estimation of KF (treated and untreated), a cost-effectiveness analysis of increasing transplantation, and a deeper investigation into province-specific barriers to KRT are needed to guide policy responses to South Africa’s growing CKD burden.

Our study has several strengths and weaknesses. Our cohort is unique in that all patients are considered transplantable when commencing on dialysis, thus confounding was reduced in the analysis of outcomes. The study initiated an electronic database for systematic collection of data on KRT referral and allocation, which is expected to enhance the quality and consistency of data reporting, particularly in category 3 patients. We were able to follow nearly all patients who were accepted onto KRT (LTFU, n = 3). The main limitation of this study was that we were not able to report true estimates of treated and untreated KF given only a select group of patients, that is, those with a probable chance of being considered for KRT, were formally referred to the unit. Regarding mortality from untreated KF, it is possible that a small number of patients would have survived for longer periods; however, the majority were referred with almost no residual kidney function and would have required dialysis imminently. There was a mismatch of timelines for records of all referred initiated in May 2013 (n = 761) and records of presented patients captured from February 2012 (n = 338). This has reduced the denominator for overall referred patients; therefore, cumulative mortality for untreated KRT is likely to be further underestimated. Finally, a number of deaths of unknown cause were recorded when not registered at a health care facility. In the author’s opinion, fluid overload was a likely cause in many of these cases.

## CONCLUSIONS

A significant majority of KF patients in the Eastern Cape public healthcare sector could not access life-sustaining KRT. Although survival posttransplantation was markedly improved compared with those remaining on dialysis, patients were more likely to die on the waiting list than receive a transplant. Focused interventions to prevent CKD and limit progression to KF, alongside increased access to transplantation, are urgently needed. Further research should determine the true burden of advanced CKD and KF, the cost-effectiveness of increasing transplantation, and assess the barriers to increasing KRT in this region as blueprint for LMIC settings.

## ACKNOWLEDGMENTS

The authors would like to thank all the participants and clinical staff involved in patient care and research at Livingstone Hospital.

## Supplementary Material


