# Supplementary material for: Transplantation Improves Patient Survival in a PD-first Program in South Africa
Source: Transplant Direct. 2026 Mar 17;12(4):e1914. doi: 10.1097/TXD.0000000000001914 (PMC12999133; doi:10.1097/TXD.0000000000001914)
Supplement: Supplementary file 1 [file txd-12-e1914-s001.pdf]

Category 3<br>Any one of the following factors excludes patients	Category 2<br>A combination of factors will progressively reduce the priority rating	Category 1<br>Patient has no Category 2 or 3 factors and satisfies all the following factors:
❑ Renal transplantation is contraindicated or carries unacceptable risks<br>❑ AIDS or HIV infection other than patients described in category 2<br>❑ Age ≥ 60 years<br>❑ Active substance abuse or dependency<br>❑ Morbid obesity (BMI 35kg/m2)<br>❑ HBeAg positive or cirrhosis<br>❑ Diabetes mellitus and aged >50 years<br>❑ Active, uncontrollable malignancy with short life expectancy<br>❑ Advanced, irreversible progressive disease of vital organs such as: Cardiac, cerebrovascular, peripheral vascular, liver, and lung disease, or unresponsive infections<br>❑ Any form of serious mental illness which results in diminished capacity for patients to take responsibility for their actions.<br>❑ Habitual non-adherence to any medical treatment	Social factors<br>❑ Poor home circumstances, including lack of access to storage space, running water, sanitation and electricity[1]<br>❑ Criminal convicted of serious offence<br>❑ Poor social network/support<br>❑ No proximity to dialysis unit<br><br>Medical factors<br>❑ Age 50 to 60 years<br>❑ BMI 30-35 kg/m2<br>❑ Hypertension with severe left ventricular dysfunction or other severe target organ damage<br>❑ HBsAg/HCV positive with no cirrhosis<br>❑ Smoking<br>❑ Diabetes mellitus<br>❑ HIV^{+} providing CD4 count >200 and undetectable viral load; if on antiretroviral (ARV) treatment, demonstrated good adherence and clinical response within 6 months.<br>❑ First presentation with ESKF requiring urgent dialysis<br>❑ Comorbid disease e.g. stable ischaemic heart disease<br>❑ Previous kidney transplant	❑ Age ≤ 50 years<br>❑ BMI less than 30 kg/m<br>❑ Gainfully employed[2]<br>❑ HIV negative<br>❑ HBsAG negative<br>❑ South African citizen

Abbreviations: HIV, human immunodeficiency virus; BMI, body mass index; Hepatitis B Surface Antigen (HBsAG); HCV, Hepatitis C virus; end-stage kidney failure, ESKF.

Notes:

[1]In a patient with poor social circumstances, PD is precluded as a treatment option and will be considered for HD if capacity.

[2]Gainfully employed refers to persons earning a salary or wage, caregiver, learners, students, self-employed individuals, seasonal workers

Reference: Moosa MR. Priority setting approach in the selection of patients in the public sector with end-stage kidney failure for renal replacement treatment in the Western Cape Province (2013) Western Cape DOH. 2021.
