# Supplementary material for: Transplantation Improves Patient Survival in a PD-first Program in South Africa
Source: Transplant Direct. 2026 Mar 17;12(4):e1914. doi: 10.1097/TXD.0000000000001914 (PMC12999133; doi:10.1097/TXD.0000000000001914)
Supplement: Supplementary file 2 [file txd-12-e1914-s002.pdf]

Table S1: Comparison of socio-demographic and clinical variables by outcome at 5 years after dialysis initiation

KRT initiation (N=136[a])	Died	Transplanted	Alive on dialysis	p-value
	N=53	N=29	N=54	
Socio-demographic				
Age	35 (29-41)	34 (29-41)	38 (28-44)	0.580
Female	21/53 (40%)	13/29 (45%)	27/54 (50%)	0.560
School Education	11 (10-12)	11 (11-12)	11 (10-12)	0.380
Employed	23/53 (43%)	12/29 (41%)	25/54 (46%)	0.900
Completed/enrolled in tertiary education	6/53 (11%)	4/29 (14%)	10/54 (19%)	0.570
Married	21/53 (40%)	16/29 (55%)	12/54 (22%)	0.009
Smoker	11/53 (21%)	2/29 (7%)	5/54 (9%)	0.130
Alcohol	11/53 (21%)	7/29 (24%)	11/54 (20%)	0.920
Illicit substance abuse	1/53 (2%)	0/29 (0%)	1/54 (2%)	-
Toilet facilities	46/53 (87%)	24/29 (83%)	40/54 (74%)	0.240
Running water	4/53 (8%)	3/29 (10%)	10/54 (19%)	0.250
Formal dwelling	50/53 (94%)	27/29 (93%)	50/54 (93%)	1.000
Clinical				
Serum creatinine	983 (676-1906)	1023 (651-1434)	1111 (683-1502)	0.970
BMI category				0.140
Normal weight	19/48 (40%)	10/28 (36%)	29/52 (56%)	
Overweight/ Obese	29/48 (60%)	18/28 (64%)	23/52 (44%)	
SBP	149 (121-180)	140 (120-168)	140 (130-170)	0.450
DBP	86 (80-98)	82 (79-98)	85 (80-98)	0.840
HIV positive	7/53 (13%)	2/29 (7%)	10/54 (19%)	0.370
Hypertension	46/53 (87%)	24/29 (83%)	46/54 (85%)	0.890
Diabetes	9/53 (17%)	2/29 (7%)	2/54 (4%)	0.053
SLE	4/53 (8%)	0/29 (0%)	2/54 (4%)	0.270
Hepatitis B	2/53 (4%)	1/29 (3%)	4/54 (7%)	0.620
Previous TB	7/53 (13%)	7/29 (24%)	7/54 (13%)	0.340

Abbreviations: BMI, body mass index; Systolic blood pressure (SBP), Diastolic blood pressure (DBP); SLE, Systemic lupus erythematosus
[a] 136 patients included in analysis of outcomes at 5 years: Of 163 allocated KRT, 4 patients died prior to initiation of KRT or not well established on KRT at 90days, 8 regained renal function, 3 patients lost to follow up, and 11 were alive on dialysis with < 5 years person time.

Table S2: Comparison of socio-demographic and clinical variables by patients on waitlist (Dialysis only) versus patients who were transplanted (Received dialysis and transplanted)

	Only received dialysis	Dialysis & Transplanted	P value
	N=117	N=34	
Age at presentation	36 (29-43)	34 (29-41)	0.390
Female	53/117 (45%)	16/34 (47%)	0.860
School Education	11 (10-12)	11 (10-12)	0.390
Employed	54/117 (46%)	15/34 (44%)	0.830
Completed/enrolled in tertiary education	19/117 (16%)	4/34 (12%)	0.600
Married	36/117 (31%)	18/34 (53%)	0.018
Smoking	18/117 (15%)	3/34 (9%)	0.410
Alcohol	27/117 (23%)	8/34 (24%)	0.960
Illicit substance abuse	3/117 (3%)	0/34 (0%)	1.000
Outside toilet facilities	21/117 (18%)	7/34 (21%)	0.730
Outside tap water source	13/117 (11%)	5/34 (15%)	0.570
Informal housing	7/117 (6%)	3/34 (9%)	0.590
Clinical variables			
Serum creatinine	1030 (660-1680)	1012 (687-1425)	0.660
BMI > 25kg/m^2	59/112 (53%)	19/31 (61%)	0.390
SBP	147 (124-180)	138 (120-164)	0.190
DBP	84 (80-97)	82 (79-96)	0.570
HIV positive	20/117 (17%)	2/34 (6%)	0.160
Hypertension	99/117 (85%)	27/34 (79%)	0.470
Diabetes	13/117 (11%)	2/34 (6%)	0.520
SLE	7/117 (6%)	0/34 (0%)	0.350
Hepatitis B	6/117 (5%)	3/34 (9%)	0.420
Previous TB	16/117 (14%)	7/34 (21%)	0.320

Abbreviations: BMI, body mass index; Systolic blood pressure (SBP), Diastolic blood pressure (DBP); SLE, Systemic lupus erythematosus

[a] 151 patients included in analysis: Of 163 allocated KRT, 4 patients died prior to initiation of KRT & 8 regained renal function
